# Effects of a Complex Care Model for Patients with Multimorbidity in Lithuania: Results from an Implementation Study

**DOI:** 10.3390/healthcare13182268

**Published:** 2025-09-11

**Authors:** Olga Vasiliauskienė, Dovydas Vasiliauskas, Aušrinė Kontrimienė, Brandon N. Jaafar, Leonas Valius, Lina Jaruševičienė, Ida Liseckienė

**Affiliations:** 1 Department of Family Medicine, Lithuanian University of Health Sciences, LT-44307 Kaunas, Lithuania; 2 Department of Chemistry, The University of Chicago, Chicago, IL 60637, USA; 3 Long School of Medicine, UT Health San Antonio, San Antonio, TX 78229, USA; 4 Department of Public Health, Lithuanian University of Health Sciences, LT-44307 Kaunas, Lithuania

**Keywords:** multimorbidity, quality of life, treatment burden, Lithuania, primary care, chronic disease, age

## Abstract

**Background/Objectives**: Multimorbidity is an increasing challenge in primary care, particularly in aging populations. The aim of the study was to evaluate the effects of a multidisciplinary, patient-centered complex care TELELISPA model on quality of life, mental health, and treatment burden among patients with multimorbidity in Lithuanian primary care settings. **Methods**: The study was conducted at seven primary healthcare centers in Lithuania and included all TELELISPA project participants who had at least two chronic health conditions including hypertension. Study participants were randomly assigned to intervention (*n* = 389) and control groups (*n* = 400): the intervention group received a coordinated, multidisciplinary care intervention for 15 months, whereas the control group received usual care. In the beginning and the end of the study, participants completed different quality of life, treatment burden and psychological health measures. **Results**: At the 15-month follow-up, the intervention group reported significantly better psychological health scores across different questionnaires—GAD-7 (*p* = 0.002), PHQ-9 (*p* < 0.001), EQ-5D-5L anxiety/depression score (*p* = 0.044), SF-36 emotional well-being score (*p* = 0.006). The intervention group also had significantly higher overall EQ-5D-5L quality of life scores (*p* = 0.02), and a better EQ-5D-5L mobility score (*p* = 0.036). Intervention group patients reported a reduced treatment burden in monitoring health conditions (*p* = 0.002), obtaining information about their conditions (*p* = 0.022), and accessing healthcare during the evenings and the weekends (*p* = 0.003). The interventions were better received by patients who were younger than 65 years of age, while mixed effects were observed between the groups with different numbers of chronic conditions, with or without polypharmacy, and different durations of education. **Conclusions**: The implementation of a multidisciplinary, team-based complex care model corresponded with improved psychological well-being, enhanced quality of life, and a partially alleviated treatment burden in patients with multimorbidity. The findings of the study support the wider adoption of integrated care models in aging populations and highlight the need for future studies assessing long-term outcomes and cost-effectiveness.

## 1. Introduction

Multimorbidity—the co-occurrence of two or more long-term medical conditions—poses a growing challenge to aging populations globally, including Lithuania [1,2]. In 2015, the prevalence of multimorbidity in Lithuania was found to range between 11% among individuals aged 45–55 and 62% among those aged 85 and older [2]. Even though 93.4% of Lithuanian patients with chronic conditions have multimorbidity, the national healthcare system largely remains based on a “one patient–one condition” model, which potentially leads to suboptimal care and the inefficient use of healthcare resources [2,3,4].

Individuals with multimorbidity in Lithuania have twice as many primary care visits, nearly ten times more home visits, and are 61% more likely to be readmitted to hospital within 30 days of discharge when compared to individuals with single chronic conditions [2]. These trends mirror those observed in other high-income countries [5,6]. Patients with multimorbidity also experience more difficulties accessing medication and specialist services and completing daily activities; they are more likely to suffer from chronic pain, mental health disorders, and poorer general health [7,8,9,10]. These combined factors place an additional financial burden on the healthcare system, resulting in more frequent home visits and higher 30-day hospital readmission rates [3]. Moreover, they significantly diminish patients’ quality of life and increase the levels of treatment burden [11,12,13,14].

The negative effects on quality of life (which typically encompasses patient mobility, ability to carry out regular tasks, mental well-being, and levels of pain) have been shown to further exacerbate physical, psychological and socioeconomic issues [15,16,17]. In the meantime, higher treatment burden (which is defined as the required effort from patients in self-managing their health and following treatment recommendations, as well as its effect on patient functioning and well-being) has been found to hinder treatment success and cause further deterioration in the quality of patients’ lives [18,19,20]. Altogether, the insufficient resources and poorer quality of healthcare dedicated to patients with multimorbidity frequently lead to a spiral of detrimental outcomes that substantially increase the risk of hospitalization and death.

Over the past decades, numerous organizational and patient-centered interventions have been introduced to improve care for patients with multimorbidity. The most effective strategies rely on patient involvement in their treatment and emphasize the holistic assessments of health needs, implemented by interdisciplinary, specially trained primary healthcare teams [21,22,23,24,25]. Complex care assessments that are specifically designed for patients with multimorbidity have been found to affect the outcomes of the type II diabetes, effectiveness of cancer diagnostics, and improve patients’ psychosocial conditions [26,27,28]. Certain interventions have been successfully tested in European countries such as Switzerland, Sweden and Spain [29,30,31], however no model has of yet been implemented in Lithuania which faces a higher-than-ever need for the holistic multimorbidity assessment and management techniques considering the ever-increasing population of patients with multimorbidity and the growing demands on the Lithuanian healthcare system.

In this publication, we present a real-world implementation study that was designed to evaluate the effects of a new complex care model in patients with multimorbidity in Lithuania. The findings indicate that the interventions corresponded with better psychological health outcomes, increased mental and physical quality of life, and diminished some aspects of treatment burden.

## 2. Materials and Methods

### 2.1. Study Design and Participants

This study involved all participants who enrolled in the TELELISPA project between August 2021 and February 2022 at seven Lithuanian primary care centers in Kaunas, Šiauliai and Tauragė municipalities (*n*  =  796). The participants were 40 to 85 years of age and had two or more long-term health conditions, with one of the conditions being arterial hypertension. Patients with mental or cognitive disorders were not eligible to participate in the study. All patients received information about the ongoing project and voluntarily signed a consent form to participate in the study.

### 2.2. Sample Size Calculation, Randomization and Group Assignment

Due to the absence of prior data, the sample size was calculated with a conservative estimate of *p* = 0.5 with a 5% margin of error (Δ = 0.05) and a 95% confidence level (α = 0.05), giving the minimum required sample size was estimated at 385 participants per group. Accounting for both intervention and control arms, the total planned sample size was 770 participants.

All patients who enrolled in the study were randomized using stratified block randomization by gender at each participating health center, with randomly permuted blocks to ensure balanced group sizes within each gender stratum. This approach ensured that men and women were evenly distributed between the intervention and control groups across all centers. Within each gender stratum, participants were randomly assigned to either the intervention or control group based on the order determined by the permuted blocks. The group assignment was carried out centrally and concealed from the study recruiters. The patients were not informed of their group assignment until they had completed the baseline surveys. Intervention-to-control contamination was deemed unlikely because the control group participants did not receive any components of the intervention and were managed separately. All patients in the intervention group were invited for a follow-up visit with the case manager and received access to other benefits of the complex care model, whereas the patients of the control group continued to receive usual care.

Although the study involved pre-assignment of groups, it was not prospectively registered as a clinical trial and should therefore be interpreted as an implementation study with a controlled design.

### 2.3. Intervention Group

**Figure 1:**
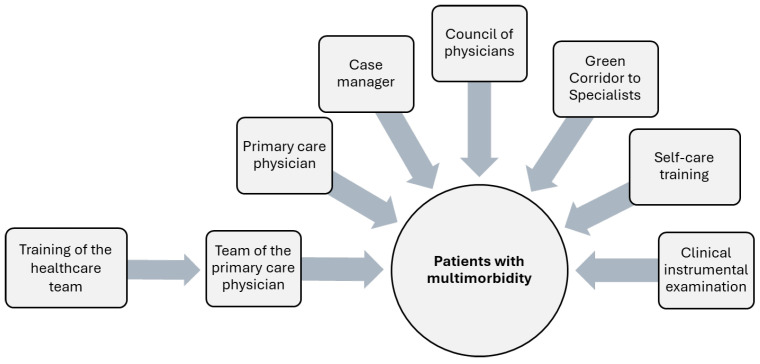
The scheme of the complex care model implementation that was applied in the study.

A structured, patient-centered care model (summarized in Figure 1) was implemented for the patients in the intervention group. Each patient was assigned a Case Manager (CM). The case manager (either a nurse or an advanced practice nurse) worked closely with the patient’s family physician. The CM conducted a comprehensive patient assessment according to the established holistic plan. The CM focused on identifying the health problems that were most important to the patient; asking about complaints, pain, function, and quality of life; screening for depression, anxiety, and dementia; evaluating risk factors, smoking, alcohol consumption and then addressing the disease-specific care with respect to the patients’ needs. The reports produced by CMs were recorded and discussed with the family physician. The collected data was considered in each of the subsequent consultations with the other healthcare specialists, and the treatment plan was reviewed and adjusted accordingly as needed.

In addition to the case manager/nurse and family physician, the extended care team included a social worker, a psychologist, a kinesiotherapist, a healthy lifestyle specialist, and an endocrinology nurse. After the review of each patient’s case by the family physician and CM, the patient was referred to the appropriate specialists within the team based on their specific needs. All of the intervention group patient cases were reviewed by a medical council which consisted of a specialist from the Lithuanian University of Health Sciences Department of Family Medicine, the patient’s family physician, and the case manager/nurse. The council discussed the clinical situations with the family physician team and determined the need for specialized examinations or priority access (“green corridor”) to specialist consultations. The intervention timeline is highlighted in Supplementary Figure S1.

The “green corridor” was an expedited pathway granting patients quicker access to specialists based on their health needs. After patients underwent laboratory and instrumental diagnostics, decisions regarding referrals were made during the medical council meetings. Remote teleconsultations were arranged with dermatologists and ophthalmologists, while in-person consultations were scheduled with cardiologists, pulmonologists, neurologists, and vascular surgeons.

### 2.4. Training of the Healthcare Teams and Patients

All healthcare team members involved in the execution of the complex care intervention received 4–6 targeted training sessions. The training sessions covered the most common pathologies seen in patients with multimorbidity, holistic care principles, multidisciplinary communication, lifestyle and preventive care counseling, as well as patient progress documentation and outcome tracking. Additional training was provided for the usage of specialized diagnostic tools such as the Holter monitors, ambulatory 24 h blood pressure monitoring devices, endocrinological assessment tools, spirometry, fundus examination via video camera, and dermatoscopy. The patients had an opportunity to attend educational sessions on healthy living, risk factor prevention and self-care practices led by a lifestyle specialist and a diabetes care specialist.

### 2.5. Outcome Measures

The intervention outcomes were determined based on study participant responses to health questionnaires when they first joined the study and again at the final visit in the 15th month of the study. At both time points, the patients completed the EQ-5D-5L quality-of-life questionnaire and the EQ visual analogue scale (EQ VAS) [32,33], the RAND Short Form 36-Item (SF-36) health survey [34], the Impact on Participation and Autonomy (IPA) questionnaire [35], the multimorbidity treatment burden questionnaire (MTBQ) [13], the GAD-7 general anxiety disorder questionnaire [36], as well as the PHQ-9 patient health questionnaire [37], and answered questions about their sociodemographic status (age, sex, duration of education, occupation). The results at the baseline and the 15-month follow-up were compiled for all patients who answered at least 75% of the MTBQ questions at the baseline (*n*  =  789, 99.1%).

### 2.6. Statistical Analysis

Statistical analysis included the non-parametric Mann–Whitney U rank-sum tests to compare the distributions of the control and treatment group patient outcomes 15 months after the baseline at α = 0.05 significance level. Aligned Rank Transformed ANOVA tests were used to determine the effects of sociodemographic and clinical factors (age, sex, work status, number of regularly taken medications, number of chronic conditions) on the control and treatment group patient outcomes at 15 months after the baseline (α = 0.05). The analysis was conducted using IBM SPSS V29.0 software and a combination of R 4.5.0 and Python 3.8 programming language scripts.

## 3. Results

**Figure 2:**
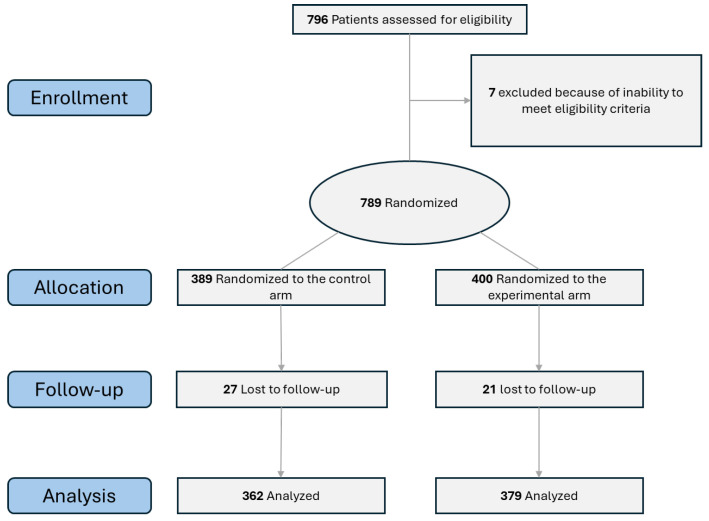
Participant flow diagram.

The study had 389 participants in the control group and 400 participants in the intervention group (Figure 2). Approximately equal numbers of participants fell into the different control and intervention groups with respect to sex, duration of education, work status, number of chronic conditions, and number of regularly taken medications at the baseline (Table 1). The study participants had a mean age of 64.4 years, women made about 60% of the study population, about half of the participants were employed, more than half of the participants had 2–5 chronic conditions, more than two thirds of the patients were regularly taking five or more medications. In the beginning of the study, the means of participant age and initial EQ-5D-5L, MTBQ, SF-36 and IPA survey response values in control and intervention groups were approximately equal and mostly not statistically significantly different (except for the difference in the initial SF-36 pain group scores which was attributed to natural sampling variability). The EQ-5D-5L questionnaire had a mean value of 0.74 out of 1.00, the MTBQ had a mean of 8.6, the GAD-7 questionnaire had a mean of 3.3 and the PHQ-9 questionnaire had a mean of 4.0. The IPA item group values ranged from 0.73 to 1.14 out of 5.0, while the SF-36 item group values ranged between 45 and 74 out of 100.

All of the study participants who had completed the baseline questionnaires were retained until the 15-month follow-up. Mann–Whitney U test results indicated that at the 15 month follow-up, patients of the intervention group had statistically significantly better scores than the patients of the control group for the following health-related quality of life outcome measures (Table 2): EQ-5D-5L overall quality-of-life score (*p* = 0.020), EQ VAS quality-of-life score (*p* = 0.012), EQ-5D-5L mobility score (*p* = 0.036), EQ-5D-5L anxiety/depression score (*p* = 0.044), SF-36 emotional well-being score (*p* = 0.006), GAD-7 general anxiety disorder score (*p* = 0.002), and PHQ-9 patient health questionnaire depression score (*p* < 0.001). Relatively big but not significant differences in outcomes between control and intervention groups were observed for the EQ-5D-5L pain/discomfort item group, and the SF-36 energy/fatigue item group. No significant differences were found for EQ-5D-5L self-care and usual activities scores, as well as SF-36 physical functioning, role limitations due to physical health, role limitations due to emotional problems, social functioning, pain and general health scores.

In terms of treatment burden and autonomy-related outcomes (Table 3) intervention group patients reported they had a significantly lower treatment burden with respect to monitoring their medical conditions (MTBQ item 5, *p* = 0.002); receiving healthcare in the evenings and at weekends (MTBQ item 9, *p* = 0.003), and obtaining clear and up-to-date information about their condition (MTBQ item 11, *p* = 0.022). Patients in the intervention group also indicated a significantly increased treatment burden for collecting prescription medication (MTBQ item 4, *p* = 0.005) and a significantly worse score for MTBQ item 12 “Making recommended lifestyle changes” (*p* = 0.007) than the control group patients. No significant differences between the intervention and control group patients were observed at the 15-month follow-up for the overall MTBQ scores and any of the IPA participation and autonomy questionnaire scores. Only a relatively large but non-significant difference was noted for the IPA work/education item group score.

**Figure 3:**
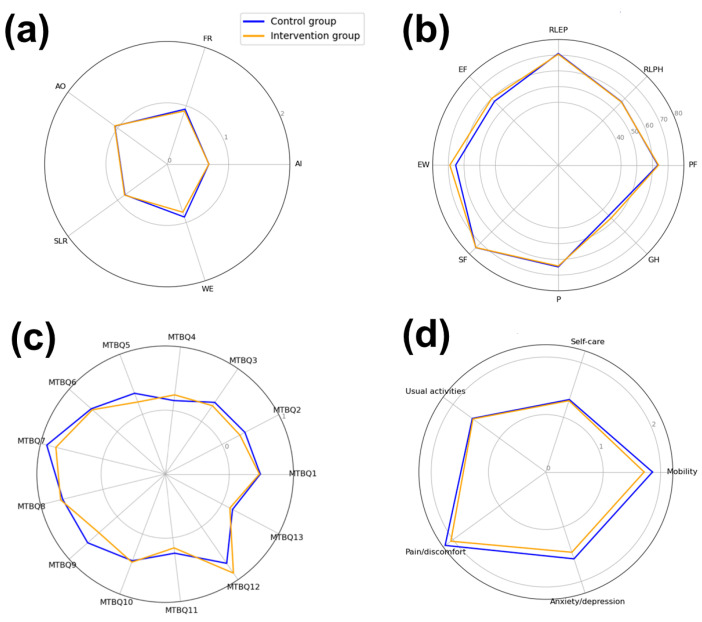
The mean scores of items in different health, quality-of-life, and treatment burden questionnaire in control group (blue) and intervention group (orange) 15 months after the start of the study. (**a**)—IPA questionnaire values, (**b**)—SF-36 questionnaire values, (**c**)—MTBQ values, (**d**)—EQ-5D-5L questionnaire values. AI—IPA autonomy indoors score; FR—IPA family role score; AO—IPA autonomy outdoors score; SLR—IPA social life and relationships score; WE—IPA work and education score. All scores range between 0 (“very good” response) to 4 (“very poor” response). SF-36 PF—SF-36 physical functioning score; SF-36 RLPH—SF-36 role limitations due to physical health score; SF-36 RLEP—SF-36 role limitations due to emotional problems score; SF-36 EF—SF-36 energy/fatigue score; SF-36 EW—SF-36 emotional well-being score; SF-36 SF—SF-36 social functioning score; SF-36 P—SF-36 pain score; SF-36 GH—SF-36 general health score. All scores range between 0 (worst possible score) and 100 (best possible score). MTBQ1–MTBQ13—scores for MTBQ items 1–13 ranging between 0 (“not difficult/no burden”) and 4 (“extremely difficult/extreme burden”). EQ M—EQ-5D-5L mobility score; EQ SC—EQ-5D-5L self-care score; EQ UA—EQ-5D-5L usual activities score; EQ PD—EQ-5D-5L pain/discomfort score; EQ AD—EQ-5D-5L anxiety/depression score. All scores range between 1 (no associated problems) and 5 (extreme problems/unable to carry out the activity).

The intervention had a bigger effect size in the items of the MTBQ and the EQ-5D-5L questionnaire, and minimal effect size in the items of the IPA questionnaire and the SF-36 questionnaire at the 15-month follow-up (Figure 3, Supplementary Table S1. The intervention group patients had notably better EQ-5D-5L scores than the control group patients for three of the EQ-5D-5L questionnaire categories—mobility (difference of −0.15), anxiety/depression (difference of −0.12) and pain discomfort (difference of −0.12) (Figure 3d). Mixed effects of the interventions were observed on different MTBQ treatment burden questionnaire items: the experimental group patients indicated a notably lower burden than the control group patients in monitoring their health conditions (difference of −0.15), seeing lots of healthcare professionals (difference of −0.15), receiving healthcare in the evenings and at the weekends (difference of −0.024) and obtaining up-to-date information about their condition (difference of −0.084) (Figure 3c). The intervention group also experienced a higher treatment burden with respect to collecting prescription medication (difference of 0.09) and making recommended lifestyle changes (difference of 0.19). The only noticeable improvement in the intervention group with respect to the SF-36 questionnaire items was observed in the SF-36 emotional well-being group (difference of 3.70) and the SF-36 energy/functioning group (difference of 2.52) (Figure 3b). No significant differences were noticeable for the IPA questionnaire item groups (Figure 3a).

More information about the time-related differences within the control and intervention groups is obtained from the summary of the health questionnaire score comparisons between the baseline and the 15-month follow up (Supplementary Table S2). The findings agree with many of the results from Table 2: the changes in values within the control and intervention groups in 15 months are significantly different for the EQ-5D-5L scale (*p* < 0.001), EQ VAS score (*p* = 0.014), EQ-5D-5L pain/discomfort score (*p* < 0.001), EQ-5D-5L anxiety/depression score (*p* = 0.021), MTBQ item 4 “collecting prescription medication” (*p* = 0.014), MTBQ item 5 “Monitoring your medical conditions” (*p* = 0.002), SF-36 energy/fatigue item group (*p* = 0.039), GAD-7 score (*p* < 0.001) and PHQ-9 score (*p* < 0.001). However, additional statistically significant differences are observed for the EQ-5D-5L mobility score (*p* = 0.044), IPA work/education score (*p* = 0.029) which had low, yet non-statistically significant *p*-values for the comparison of the control and intervention group scores at the 15-month follow-up. This suggests that the observed between-group differences at 15 months are not due to baseline imbalances, and that the intervention effect is robust and that the results are internally consistent.

Additional insight into the differences in health outcomes at the 15-month follow-up is available from the summary of the effects of sociodemographic and clinical factors on the health score distributions in control and intervention groups (Table 4). The ART ANOVA results show how the distribution of the scores of items in different questionnaires varied in study participant groups of different ages, groups with different number of chronic conditions, number of regularly taken medications, and a different duration of education. First, the study participants who were younger than 65 years of age received the interventions had a statistically significant improvement in SF-36 energy/fatigue, emotional well-being, general health and physical health scores, whereas the patients who were older than 65 years of age did not have an improvement as stark as that for the respective scores. The participants who were younger than 65 years of age and received the intervention also reported an improvement with the burden of taking lots of medications, monitoring their medical conditions and seeing lots of different health professionals—patients older than 65 years of age had a similar but less pronounced improvement for monitoring health conditions and seeing health professionals, and a worsening score for the burden of taking many medications. The patients younger than 65 years of age who received the intervention had an improvement in the autonomy outdoors and social life and relationships scores, whereas the patients who were older than 65 years of age and received the intervention indicated the opposite effect—worse autonomy outdoors and worse social life and relationships. The patients who were younger than 65 years of age also had an improvement in GAD-7 composite scores (mean decrease of 1.68) and an improvement in PHQ-9 composite scores (mean decrease of 1.72), whereas the patients who were older than 65 years of age had less notable decrease in mean values for respective scores. Altogether, the results suggest that the interventions generally had a more positive effect in younger patients with respect to their treatment burden as well as the physical and mental health than in older patients.

The interventions also corresponded with an overall improvement in health outcomes among patients with five or fewer chronic conditions—they had noticeably better SF-36 general health, pain, physical health scores, as well as IPA work and education and social life and relationships scores than individuals with more than five conditions. At the 15-month follow-up, the intervention group patients with more than five conditions had better emotional well-being and anxiety/depression scores, as well as better treatment burden outcomes with respect to remembering taking medication and seeing lots of health professionals; however, they reported an increased burden in collecting prescription medication and monitoring their health conditions. The patients with polypharmacy (five or more regularly taken medications) who received interventions reported better EQ-5D-5L anxiety/depression scores, PHQ-9 depression scores and role limitation due to emotional problem scores than patients without polypharmacy. Polypharmacy patients also had higher treatment burden with respect to collecting prescription medication and paying for medications and treatment than the non-polypharmacy patients. Patients with 14 or more years of education who underwent the interventions had significantly higher SF-36 emotional well-being scores and PHQ-9 composite scores than patients with fewer than 14 years of education. Overall, the findings show that the interventions had heterogenous effects across subgroups of patients with multimorbidity who had different sociodemographic and clinical characteristics.

## 4. Discussion

The findings of the study indicate that the 15-month personalized complex care intervention had a measurable helpful effect on Lithuanian patients with multimorbidity. Compared to the patients of the control group, the patients who received the interventions reported an improved mental and physical health, a better overall quality of life, and reductions in key domains of treatment burden—outcomes which are all relevant for designing health services that are better aligned with the complex needs of individuals living with multiple chronic conditions.

The most significant improvement in the intervention group was seen in the domain of psychological health. In agreement with previous findings [25,38], the patients who received intervention reported better scores in each of the mental/emotional health questionnaires at the 15-month follow-up. The mental health measures showed the most pronounced response to the intervention among all questionnaire domains, with effect sizes of 0.354 for GAD-7 questionnaire, 0.574 for PHQ-9 questionnaire, 0.291 for the SF-36 emotional well-being group, and 0.150 for the EQ-5D-5L anxiety/depression item group. These findings support the current evidence that multidisciplinary care models, particularly those involving nursing staff and mental health professionals, enhance emotional support and continuity of care, and contribute to better psychological outcomes [22].

Another important effect of the implementation of the complex care plan was seen with respect to the quality of life. The intervention group had statistically significant improvements in the overall EQ-5D-5L quality-of-life questionnaire scores, the EQ VAS score, the EQ-5D-5L domain of mobility and, arguably, an improvement in the EQ-5D-5L pain/discomfort domain. Similarly to previously published studies [39,40] the quality-of-life measures had lower effect sizes (0.12–0.23) than the mental health questionnaire items despite their statistical significance across multiple time point comparisons and group-level distributions. Mobility and pain/discomfort domains are particularly relevant in older adults whose functional limitations and chronic pain significantly contribute to decreased independence and place a substantial burden on healthcare systems [41,42]. Addressing physical functioning and pain through holistic care may help interrupt the cycle of disability, treatment nonadherence and psychological decline.

Additionally, the intervention group experienced notable improvement with respect to different facets of treatment burden. Based on the results of the MTBQ, patients who received interventions had more clarity of health information, more convenient condition monitoring, and easier access to care during evenings and weekends. These findings align with literature that highlights the importance of coordinated care in reducing the frequent complexity and fragmentation of chronic disease management [43]. While the interventions led to a reduction in certain areas of treatment burden, they did not reduce the overall treatment burden scores and exacerbated the burden of collecting prescription medication and making the recommended lifestyle changes in agreement to previous studies [44]. The few negative effects on treatment burden are expected as the intervention group had more appointments with the healthcare specialists which may result in more prescriptions and more complex lifestyle-related recommendations—a comprehensive reduction in all aspects of treatment burden in the future studies may require targeted interventions beyond the scope of the primary care.

The interventions had divergent effects on patients in different sociodemographic groups. In agreement to previous findings [45], the holistic healthcare plan was better received by patients who were younger than 65 years of age, as they reported a more pronounced improvement with respect to energy/fatigue, emotional well-being, general health, physical health, autonomy outdoors, social life and relationships, depression, anxiety, as well as less burden in seeing health specialists and monitoring health conditions than patients who were older than 65 years of age. The interventions were arguably received more poorly by the older patients who, in general, tend to have more serious mobility issues and more complex chronic disorders [30,31]—the older patients might benefit from more optimal scheduling of medication regimen and medical specialist appointments as these were important factors contributing to increased treatment burden. In terms of the number of chronic disorders, the interventions corresponded with significantly higher improvement in general health, physical health, and social life and relationships for patients who had five or fewer chronic conditions yet contributed substantially more to better emotional well-being and anxiety/depression for those who had more than five conditions. The intervention group patients with more than five disorders reported a higher treatment burden in collecting prescription medication, paying for treatment and monitoring their health conditions. Finally, the intervention positively affected patients with polypharmacy (concurrent use of five or more medications), as they reported diminished depression and fewer emotional problems whereas these effects were not observed among patients who regularly consumed fewer than five medications. These results suggest that future interventions can be tailored to individuals within different sociodemographic and health groups via patient priorities care approaches [46] to maximize the positive intervention effects.

### Strengths and Limitations

This study has several key strengths. It employed a randomized group allocation in a real-world implementation context with a large, demographically diverse sample representative of the Lithuanian population of patients with multimorbidity. The findings may be cautiously generalizable to the Baltic countries and other Eastern-Middle European countries with similar social and cultural demographics. The interventions included a unique complex care approach that included a case manager, physical examinations, direct access to specialist physicians, and the team of a family physician, as well as the continued follow-ups by a board of healthcare specialists. This study used validated outcome measures across psychosocial, physical, and functional domains and indicated multiple statistically significant relationships between the implementation of the interventions and changes in health outcomes. Additionally, the study compared the effects of the healthcare interventions across different sociodemographic and health groups (age, number of chronic conditions, number of taken medications, duration of education) which is an underexplored area in studies of similar scope.

However, there are also limitations. Despite multiple significant intervention effects, most of them had small-to-moderate effect sizes (approximately 0.15–0.35). Some findings may not have remained significant under more conservative correction methods for multiple comparisons, such as the Bonferroni adjustment. Some bias could have come from the exclusion of patients with psychiatric disorders, even though it is a typical decision in similar studies [47]. The study also did not consider the number of health specialist visits, did not evaluate the physical activity of the participants, and was started with COVID-19 restrictions still in place. The study’s multi-component design precluded analysis of the individual effects of each intervention element. The duration of the follow-up (15 months) may also not be sufficient to capture all longer-term impacts on health-related quality of life or healthcare utilization. The improvement of the psychological health status could have been biased by the increased number of medical professional visits and the received help for coping with the chronic disorders or treatment burden. Additional bias could have been introduced due to inter-group contamination if control group participants decided at any point in the study to independently pursue additional appointments with the healthcare professionals, even though no record of such activity was obtained. Moreover, no economic evaluation was conducted, which restricts the applications of this study in policy-level decision-making. Future studies would be needed to clarify the long-term effectiveness and cost-efficiency of the interventions and to identify which components contribute most meaningfully to patient outcomes.

Ultimately, the study was not prospectively recorded in a clinical trial registry and should therefore be viewed as a randomized implementation evaluation rather than a classical randomized controlled trial. This decision was based on the authors’ original interpretation that the intervention—being non-invasive, behavioral, and delivered within the context of routine care—did not fall under the formal definition of a clinical trial at the time of study design. We affirm that the study was conducted with full ethical approval, randomization procedures, and a pre-specified analysis plan, ensuring methodological transparency and rigor.

## 5. Conclusions

This study demonstrates that a 15-month implementation of a complex care model for patients with multimorbidity in Lithuania can significantly improve mental health and overall quality of life. The strongest positive effects were observed in anxiety, depression, and emotional well-being measures. The intervention reduced the treatment burden in several areas, including condition monitoring and access to care, although it increased burden related to medication collection and lifestyle changes. Benefits of the intervention were more pronounced in younger patients (<65 years) and those with fewer chronic conditions, suggesting that intervention approaches tailored specifically to certain sociodemographic groups could be implemented in the future to optimize the care for patients with multimorbidity. Future adaptations of the intervention should also consider differences across clinical contexts and healthcare systems, while aligning with patient priority care approaches, to ensure successful implementation and relevance in diverse settings.

## Figures and Tables

**Table 1:** Baseline characteristics of sociodemographic data and questionnaire scores.

	Control Group (N = 389)	Intervention Group (N = 400)
Mean age, years (SE) *	64.7 (0.54)	64.1 (0.47)
Sex		
Female	235 (60.4%)	246 (61.5%)
Male	154 (39.6%)	154 (38.5%)
Duration of education		
<14 years	286 (73.9%)	274 (69.0%)
≥14 years	101 (26.1%)	123 (31.0%)
Work status		
Employed	175 (45.0%)	185 (46.3%)
Retired/Unemployed/Other	214 (55.0%)	215 (53.8%)
Number of chronic conditions		
2–5	224 (57.6%)	205 (51.2%)
>5	165 (42.4%)	195 (48.8%)
Number of regularly taken medications		
<5	134 (34.4%)	111 (27.8%)
≥5	255 (65.6%)	289 (72.2%)
Questionnaire scores **		
EQ-5D-5L score	0.75 (0.009); *n* = 388	0.73 (0.009); *n* = 399
MTBQ score	8.69 (0.45); *n* = 389	8.55 (0.40); *n* = 400
IPA scores		
IPA AI	0.73 (0.036); *n* = 389	0.74 (0.03); *n* = 399
IPA FR	0.96 (0.039); *n* = 389	0.96 (0.03); *n* = 399
IPA AO	1.14 (0.039); *n* = 389	1.17 (0.04); *n* = 399
IPA SLR	0.90 (0.033); *n* = 389	0.90 (0.03); *n* = 399
IPA WE	0.93 (0.03); *n* = 389	0.95 (0.03); *n* = 399
SF-36 scores		
SF-36 PF	65.13 (1.28); *n* = 380	63.18 (1.19); *n* = 394
SF-36 RLPH	55.99 (2.26); *n* = 377	54.54 (2.15); *n* = 391
SF-36 RLEP	72.77 (2.10); *n* = 377	72.05 (1.98); *n* = 390
SF-36 EF	57.21 (0.91); *n* = 380	56.73 (0.92); *n* = 394
SF-36 EW	67.54 (0.92); *n* = 381	68.35 (0.84); *n* = 394
SF-36 SF	74.13 (1.11); *n* = 381	73.47 (1.11); *n* = 394
SF-36 P	64.17 (1.31); *n* = 381	59.85 (1.09); *n* = 394
SF-36 GH	45.45 (0.81); *n* = 380	45.78 (0.83); *n* = 394
GAD-7 score	3.06 (0.17); *n* = 388	3.64 (0.21); *n* = 399
PHQ-9 score	4.02 (0.19); *n* = 360	3.98 (0.20); *n* = 374

* SE—standard error. ** reported as “Mean (Standard error); sample size”. EQ-5D-5L score—values from the EQ-5D-5L utility value scale where −0.59 corresponds to the worst possible life quality state and 1.00 corresponds to the best life quality state. MTBQ score—values from the MTBQ numeric scale ranging between 0 (no treatment burden) and 100 (maximum treatment burden) calculated as mean(MTBQ item values) × 25. IPA scores—values that correspond to the Impact on Participation and Autonomy questionnaire item groups. IPA AI—IPA autonomy indoors score; IPA FR—IPA family role score; IPA AO—IPA autonomy outdoors score; IPA SLR—IPA social life and relationships score; IPA WE—IPA work and education score. All scores range between 0 (“very good” response) to 4 (“very poor” response). SF-36 scores—values that correspond to the RAND Short Form 36-item questionnaire item groups. SF-36 PF—SF-36 physical functioning score; SF-36 RLPH—SF-36 role limitations due to physical health score; SF-36 RLEP—SF-36 role limitations due to emotional problems score; SF-36 EF—SF-36 energy/fatigue score; SF-36 EW—SF-36 emotional well-being score; SF-36 SF—SF-36 social functioning score; SF-36 P—SF-36 pain score; SF-36 GH—SF-36 general health score. All SF-36 scores range between 0 (worst possible score) and 100 (best possible score). GAD-7 score—General Anxiety Disorder-7 score where 0 corresponds to minimal anxiety and 21 corresponds to severe anxiety. PHQ-9 score—Patient Health Questionnaire-9 score where 0 corresponds to minimal depression and 27 corresponds to severe depression.

**Table 2:** Summary of the health-related quality of life, anxiety and depression outcomes (EQ-5D-5L, SF-36, GAD-7 and PHQ-9 questionnaire scores) at the 15-month follow-up.

	Control Group Median (IQR)	Intervention Group Median (IQR)	Mann–Whitney U Test *p*-Value	Effect Size *
EQ-5D-5L questionnaire				
EQ-5D-5L score	0.739 (0.19); *n* = 357	0.767 (0.18); *n* = 378	*p* = **0.020**	0.195
EQ VAS score	60.0 (25.0); *n* = 358	70.0 (30.0); *n* = 381	*p* = **0.012**	0.234
EQ-5D-5L mobility score	2.0 (1.0); *n* = 357	1.0 (1.0); *n* = 378	*p* = **0.036**	0.163
EQ-5D-5L self-care score	1.0 (1.0); *n* = 357	1.0 (0.0); *n* = 378	*p* = 0.30	0.040
EQ-5D-5L usual activities score	1.0 (1.0); *n* = 357	1.0 (1.0); *n* = 378	*p* = 0.56	0.013
EQ-5D-5L pain/discomfort score	2.0 (1.0); *n* = 357	2.0 (2.0); *n* = 378	*p* = 0.068	0.123
EQ-5D-5L anxiety/depression score	2.0 (1.0); *n* = 357	1.0 (1.0); *n* = 378	*p* = **0.044**	0.150
SF-36 questionnaire				
SF-36 PF	70.0 (35.0); *n* = 357	65.0 (30.0); *n* = 377	*p* = 0.77	0.003
SF-36 RLPH	75.0 (100.0); *n* = 357	75.0 (75.0); *n* = 373	*p* = 0.92	0.000
SF-36 RLEP	100.0 (66.7); *n* = 356	100.0 (66.7); *n* = 372	*p* = 0.63	0.009
SF-36 EF	60.0 (20.0); *n* = 357	60.0 (25.0); *n* = 376	*p* = 0.059	0.132
SF-36 EW	68.0 (28.0); *n* = 358	72.0 (24.0); *n* = 375	*p* = **0.006**	0.291
SF-36 SF	77.5 (32.5); *n* = 357	77.5 (32.5); *n* = 374	*p* = 0.82	0.002
SF-36 P	67.5 (35.0); *n* = 357	67.5 (32.5); *n* = 373	*p* = 0.50	0.013
SF-36 GH	45.0 (20.0); *n* = 357	45.0 (25.0); *n* = 377	*p* = 0.15	0.076
GAD-7 score	3.0 (5.0); *n* = 360	2.0 (4.0); *n* = 377	*p* = **0.002**	0.354
PHQ-9 score	4.0 (4.0); *n* = 360	2.0 (4.0); *n* = 374	*p* < **0.001**	0.574

* Effect size is calculated using the standardized effect size formula r = z/sqrt(n) where z is the z-score from the Mann–Whitney U test obtained from the continuity correction and n is the total sample size across the control and intervention groups. At the baseline, the questionnaire was completed by 389 participants in the control group and 400 participants in the intervention group. EQ-5D-5L score—values from EQ-5D-5L utility value scale where −0.59 corresponds to the worst possible life quality state and 1.00 corresponds to the best life quality state. EQ VAS score—values of the EQ visual analogue scale ranging between 0 (worst self-rated health score) and 100 (best self-rated health score). All scores range between 1 (no associated problems) and 5 (extreme problems/unable to carry out the activity). SF-36 PF—SF-36 physical functioning score; SF-36 RLPH—SF-36 role limitations due to physical health score; SF-36 RLEP—SF-36 role limitations due to emotional problems score; SF-36 EF—SF-36 energy/fatigue score; SF-36 EW—SF-36 emotional well-being score; SF-36 SF—SF-36 social functioning score; SF-36 P—SF-36 pain score; SF-36 GH—SF-36 general health score. All scores range between 0 (worst possible score) and 100 (best possible score). GAD-7 score—General Anxiety Disorder-7 score where 0 corresponds to minimal anxiety and 21 corresponds to severe anxiety. PHQ-9 score—Patient Health Questionnaire-9 score where 0 corresponds to minimal depression and 27 corresponds to severe depression. Mann-Whitney *p*-values highlighted in bold correspond to statistically significant entries at significance level α=0.05.

**Table 3:** Summary of the treatment burden and autonomy outcomes (MTBQ and IPA questionnaire scores) at the 15-month follow-up.

	Control Group Median (IQR)	Intervention Group Median (IQR)	Mann–Whitney U Test *p*-Value	Effect Size *
MTBQ				
MTBQ score	7.69 (13.5); *n* = 362	7.69 (13.5); *n* = 379	*p* = 0.62	0.009
MTBQ wo 9/10 score **	6.82 (13.6); *n* = 362	6.82 (11.4); *n* = 379	*p* = 0.77	0.0002
MTBQ item 1	0.0 (1.0); *n* = 343	0.0 (1.0); *n* = 364	*p* = 0.85	0.001
MTBQ item 2	0.0 (1.0); *n* = 353	0.0 (0.0); *n* = 370	*p* = **0.041**	0.155
MTBQ item 3	0.0 (0.0); *n* = 356	0.0 (0.0); *n* = 375	*p* = 0.81	0.002
MTBQ item 4	0.0 (0.0); *n* = 357	0.0 (0.0); *n* = 376	*p* = **0.005**	0.296
MTBQ item 5	0.0 (1.0); *n* = 357	0.0 (0.0); *n* = 375	*p* = **0.002**	0.344
MTBQ item 6	0.0 (1.0); *n* = 354	0.0 (1.0); *n* = 377	*p* = 0.87	0.001
MTBQ item 7	1.0 (1.0); *n* = 352	0.0 (1.0); *n* = 372	*p* = 0.065	0.126
MTBQ item 8	0.0 (1.0); *n* = 345	0.0 (1.0); *n* = 368	*p* = 0.90	0.001
MTBQ item 9 #	0.0 (1.0); *n* = 234	0.0 (1.0); *n* = 242	*p* = **0.003**	0.402
MTBQ item 10 #	0.0 (1.0); *n* = 254	0.0 (1.0); *n* = 263	*p* = 0.57	0.014
MTBQ item 11	0.0 (0.0); *n* = 355	0.0 (0.0); *n* = 372	*p* = **0.022**	0.195
MTBQ item 12	0.0 (1.0); *n* = 355	1.0 (2.0); *n* = 376	*p* = **0.007**	0.274
MTBQ item 13	0.0 (0.0); *n* = 353	0.0 (0.0); *n* = 371	*p* = 0.12	0.088
IPA questionnaire				
IPA AI	0.571 (1.0); *n* = 360	0.571 (1.0); *n* = 378	*p* = 0.96	0.000
IPA FR	1.0 (1.14); *n* = 360	1.0 (1.29); *n* = 378	*p* = 0.80	0.002
IPA AO	1.0 (1.0); *n* = 360	1.0 (1.2); *n* = 378	*p* = 0.63	0.008
IPA SLR	0.857 (1.0); *n* = 360	1.0 (1.0); *n* = 378	*p* = 0.70	0.005
IPA WE	0.833 (0.67); *n* = 356	0.667 (0.83); *n* = 377	*p* = 0.12	0.085

* Effect size is calculated using the standardized effect size formula r = z/sqrt(n) where z is the z-score from the Mann–Whitney U test obtained from the continuity correction and n is the total sample size across the control and intervention groups. At the baseline, the questionnaire was completed by 389 participants in the control group and 400 participants in the intervention group. EQ-5D-5L score—values from EQ-5D-5L utility value scale where −0.59 corresponds to the worst possible life quality state and 1.00 corresponds to the best life quality state. MTBQ score—values from the MTBQ numeric scale ranging between 0 (no treatment burden) and 100 (maximum treatment burden) calculated as mean(MTBQ item values) × 25. ** MTBQ wo 9/10 score—values from the MTBQ numeric scale after the exclusion of responses for MTBQ items 9 and 10. MTBQ item 1–MTBQ item 13—scores for MTBQ items 1–13 ranging between 0 (“not difficult/no burden”) and 4 (“extremely difficult/extreme burden”). Item 1—“Taking lots of medications”, item 2—“Remembering how and when to take medication”, item 3—“Paying for medications and treatment”, item 4—“Collecting prescription medication”, item 5—“Monitoring your medical conditions”, item 6—“Arranging appointments with health professionals”, item 7—“Seeing lots of different health professionals”, item 8—“Getting time off work, arranging transport, etc., to see doctors”, item 9—“Getting healthcare in the evenings and at weekends”, item 10—“Getting help from community services”, item 11—“Obtaining clear and up-to-date information about your condition”, item 12—“Making recommended lifestyle changes”, item 13—“Having to rely on help from family and friends”. # In the calculation of the 13-item MTBQ scores, items 9 and 10 were assigned values of zero. IPA AI—IPA autonomy indoors score; IPA FR—IPA family role score; IPA AO—IPA autonomy outdoors score; IPA SLR—IPA social life and relationships score; IPA WE—IPA work and education score. All scores range between 0 (“very good” response) to 4 (“very poor” response). Mann-Whitney *p*-values highlighted in bold correspond to statistically significant entries at significance level α = 0.05.

**Table 4:** Summary of questionnaire items that have statistically significant differences in value distributions between control and intervention groups across different categories of sociodemographic and clinical characteristics. The non-empty table value pairs correspond to the difference in scores between the intervention and control groups at the 15-month follow-up in each of the categorical variable subcategories that had a statistically significant *p*-value at α = 0.05 using the ART ANOVA test.

Questionnaire Item/Group *	Age		Number of Chronic Conditions		Number of Medications		Education	
	<65	≥65	≤5	>5	<5	≥5	≥14 years	<14 years
SF-36 ^a^								
SF-36 energy/fatigue	5.58 *	0.00 *	–	–	–	–	–	–
SF-36 emotional well-being	6.75	1.04	2.13	5.97	–	–	5.92	−2.35
SF-36 general health	4.96	−0.36	4.70	0.13	–	–	–	–
SF-36 pain	–	–	2.31	−3.45	–	–	−2.35	3.87
SF-36 role limitations due to physical health	8.04	−4.38	4.53	−3.75	–	–	0.32	−0.67
SF-36 role limitations due to emotional problems	–	–	–	–	−3.33	1.520	1.07	−3.66
MTBQ ^b^								
MTBQ item 1	−0.11	0.13	–	–	–	–	–	–
MTBQ item 2	–	–	−0.04	−0.16	–	–	–	–
MTBQ item 3	–	–	–	–	−0.073	0.070	−0.068	−0.03
MTBQ item 4	–	–	0.02	0.15	0.002	0.121	–	–
MTBQ item 5	−0.22	−0.09	−0.10	0.21	–	–	−0.19	−0.04
MTBQ item 7	−0.21	−0.08	−0.01	−0.36				
EQ-5D-5L ^c^								
EQ-5D-5L self-care	–	–	–	–	–	–	−0.04	0.04
EQ-5D-5L anxiety/depression	–	–	−0.06	−0.22	0.021	−0.193	–	–
IPA ^d^								
IPA autonomy outdoors	−0.08	0.11	–	–	–	–	–	–
IPA social life and relationships	−0.13	0.20	−0.08	0.08	–	–	−0.02	0.11
IPA work and education	–	–	−0.19	0.03	–	–	–	–
GAD-9 score ^e^	−1.68	−0.05	–	–	–	–	–	–
PHQ-9 score ^e^	−1.72	−0.27	–	–	−0.10	−1.465	−1.45	0.19

* Calculation example. The distributions of SF-36 energy/fatigue item group scores between intervention and control group subjects with respect to age categories (<65 years of age and ≥65 years of age) were found to be statistically different with *p* = 0.00758 using the ART ANOVA test (the distributions are depicted in Supplementary Figure S2). The difference in SF-36 energy/fatigue values between the intervention and control group mean scores was 5.584 for the <65-year age group and 0.00 for the ≥65-year age group, which suggests that intervention contributed to higher energy in younger patients and had no noticeable effect for the older patients. Hyphens (–) correspond to empty value pairs correspond to questionnaire items that did not have statistically significant ART ANOVA test *p*-values between the intervention and control groups with respect to certain categorical variables. Questionnaire and questionnaire items that did not have statistically significant ART ANOVA *p*-values for any of the represented categorical variables (age, number of chronic conditions, number of medications, education duration) are not included in the table. ^a^ Positive SF-36 item group difference corresponds to an intervention-related improvement, and negative scores indicate intervention-related worsening values. The net values which were used for the calculation of the difference ranged between 0 (worst score) and 100 (best score). ^b^ Positive MTBQ item difference corresponds to intervention-related worsening values, and negative scores indicate an intervention-related improvement. The net values which were used for the calculation of the difference ranged between 0 (best score) and 4 (worst score). ^c^ Positive EQ-5D-5L item difference corresponds to intervention-related worsening values, and negative scores indicate an intervention-related improvement. The net values which were used for the calculation of the difference ranged between 1 (best score) and 5 (worst score). ^d^ Positive IPA item group difference corresponds to intervention-related worsening values, and negative scores indicate an intervention-related improvement. The net values which were used for the calculation of the difference ranged from 0 (best score) to 4 (best score). ^e^ Positive GAD-7 and PHQ-9 item group difference corresponds to an intervention-related worsening values, and negative scores indicate intervention-related improvement. The net values of GAD-7 which were used for the calculation of the difference ranged from 0 (best score) to 21 (worst score). The net values of PHQ-9 which were used for the calculation of the difference ranged from 0 (worst score) to 27 (best score).

## Data Availability

The data are available upon reasonable request and with appropriateethical approvals.

## Supplementary Materials

The following supporting information can be downloaded at: https://www.mdpi.com/article/10.3390/healthcare13182268/s1, Supplementary Table S1. Summary of study outcome means at 15 months. Supplementary Table S2. Change in different quality-of-life measure scores between baseline and the 15-month follow-up. Supplementary Figure S1. Intervention timeline. The intervention group received a more extensive examination and care than is common in typical clinical practice over a span of 15 months. The intervention consisted of a holistic assessment of patient health and the creation of a personalized healthcare plan by the patient case manager and a primary care physician (months 1-5, A), an additional patient care assessment by a board of primary care specialists (months 4-5, B), the execution of the holistic healthcare plan (months 6-12, C), additional personalized expedited need-based examinations (months 6-9, D) and an ultimate assessment of the success in executing the holistic personalized healthcare plan (months 13-15, E). Supplementary Figure S2. Example distribution of different health outcome scores that are statistically significantly different among control and intervention group patients in different categories of sociodemographic or clinical factor variables. Images on the left—mean and standard error values, images on the right—boxplots with highlighted median, interquartile range, ±1.5 × IQR values and outliers. Control group – blue, intervention group—gold. The statistical significance of the differences in distributions was calculated with ANOVA and ART ANOVA methods, results are reported in Table 3. Only the distributions that have *p* < 0.05 values for both the ANOVA and ART ANOVA tests are reported in Table 3 and in this figure.
