# Supplementary material for: Effects of a Complex Care Model for Patients with Multimorbidity in Lithuania: Results from an Implementation Study
Source: Healthcare (Basel). 2025 Sep 11;13(18):2268. doi: 10.3390/healthcare13182268 (PMC12469520; doi:10.3390/healthcare13182268)
Supplement: Supplementary file 1 [file healthcare-13-02268-s001.zip › healthcare-3744347-supplementary.pdf]

Supplementary Information Appendix

Supplementary Table S1. Summary of study outcome means at 15 months.

	Control group (N=389)	Intervention group (N=400)
EQ-5D-5L questionnaire		
EQ-5D-5L score	0.73 (0.01); n=357	0.76 (0.01); n=378
EQ VAS score	62.6 (0.87); n=358	65.5 (0.91) n=381
EQ-5D-5L mobility score	1.86 (0.05); n=357	1.71 (0.05); n=378
EQ-5D-5L self-care score	1.33 (0.03); n=357	1.30 (0.03); n=378
EQ-5D-5L usual activities score	1.59 (0.04); n=357	1.57 (0.04); n=378
EQ-5D-5L pain/discomfort score	2.17 (0.05); n=357	2.05 (0.05); n=378
EQ-5D-5L anxiety/ depression score	1.59 (0.04); n=357	1.47 (0.04); n=378
MTBQ questionnaire		
MTBQ score	10.59 (0.59); n=362	9.90 (0.52); n=379
MTBQ wo 9/10 score	9.97 (0.56); n=362	9.72 (0.51); n=400
MTBQ item 1	0.48 (0.04); n=343	0.47 (0.04); n=364
MTBQ item 2	0.41 (0.04); n=353	0.32 (0.03); n=370
MTBQ item 3	0.36 (0.05); n=356	0.30 (0.04); n=375
MTBQ item 4	0.16 (0.03); n=357	0.25 (0.03); n=376
MTBQ item 5	0.35 (0.04); n=357	0.21 (0.03); n=375
MTBQ item 6	0.54 (0.04); n=354	0.52 (0.04); n=377
MTBQ item 7	0.90 (0.06); n=352	0.76 (0.05); n=372
MTBQ item 8	0.65 (0.05); n=345	0.68 (0.05); n=368
MTBQ item 9	0.62 (0.06); n=234	0.38 (0.05); n=242
MTBQ item 10	0.45 (0.05); n=254	0.47 (0.05); n=263
MTBQ item 11	0.24 (0.03); n=355	0.16 (0.02); n=372
MTBQ item 12	0.69 (0.05); n=355	0.88 (0.05); n=376
MTBQ item 13	0.19 (0.03); n=353	0.14 (0.03); n=371
IPA questionnaire		
IPA AI	0.68 (0.03); n=360	0.68 (0.03); n=378
IPA FR	0.94 (0.04); n=360	0.91 (0.04); n=378
IPA AO	1.06 (0.04); n=360	1.06 (0.04); n=378
IPA SLR	0.85 (0.03); n=360	0.86 (0.03); n=378
IPA WE	0.91 (0.04); n=356	0.82 (0.03); n=377
SF-36 questionnaire		
SF-36 PF	63.5 (1.35); n=357	63.9 (1.23); n=377
SF-36 RLPH	57.0 (2.37); n=357	56.8 (2.16); n=373

SF-36 RLEP	71.1 (2.23); n=356	70.6 (2.08); n=372
SF-36 EF	57.5 (0.86); n=357	60.0 (0.87); n=376
SF-36 EW	65.4 (0.92); n=358	69.1 (0.80); n=375
SF-36 SF	74.3 (1.04); n=357	74.2 (1.07); n=374
SF-36 P	64.8 (1.22); n=357	64.2 (1.14); n=373
SF-36 GH	45.4 (0.81); n=357	47.6 (0.84); n=377
GAD-7 score	3.46 (0.21); n=360	2.69 (0.18); n=377
PHQ-9 score	4.51 (0.22); n=360	3.52 (0.20); n=374

EQ-5D-5L score – values from EQ-5D-5L utility value scale where -0.59 corresponds to the worst possible life quality state and 1.00 corresponds to the best life quality state
MTBQ score – values from MTBQ numeric scale ranging between 0 (no treatment burden) and 100 (maximum treatment burden) calculated as mean(MTBQ questionnaire item values) × 25. **MTBQ wo 9/10 score – values from the MTBQ numeric scale after the exclusion of responses for MTBQ items 9 and 10
EQ VAS score – values of the EQ visual analogue scale ranging between 0 (worst self-rated health score) and 100 (best self-rated health score). All scores range between 1 (no associated problems) and 5 (extreme problems/unable to carry out the activity)
MTBQ item 1-MTBQ item 13 – scores for MTBQ questionnaire items 1-13 ranging between 0 (“not difficult/no burden”) and 4 (“extremely difficult/extreme burden”). Item 1 – “Taking lots of medications”, item 2 – “Remembering how and when to take medication”, item 3 – “Paying for medications and treatment”, item 4 – “Collecting prescription medication”, item 5 – “Monitoring your medical conditions”, item 6 – “Arranging appointments with health professionals”, item 7 – “Seeing lots of different health professionals”, item 8 – “Getting time off work, arranging transport, etc. to see doctors”, item 9 – “Getting health care in the evenings and at weekends”, item 10 – “Getting help from community services”, item 11 – “Obtaining clear and up-to-date information about your condition”, item 12 – “Making recommended lifestyle changes”, item 13 – “Having to rely on help from family and friends”. IPA AI – IPA autonomy indoors score; IPA FR – IPA family role score; IPA AO – IPA autonomy outdoors score; IPA SLR – IPA social life and relationships score; IPA WE – IPA work and education score. All scores range between 0 (“very good” response) to 4 (“very poor” response). SF-36 PF – SF-36 physical functioning score; SF-36 RLPH – SF-36 role limitations due to physical health score; SF-36 RLEP – SF-36 role limitations due to emotional problems score; SF-36 EF – SF-36 energy/fatigue score; SF-36 EW – SF-36 emotional well-being score; SF-36 SF – SF-36 social functioning score; SF-36 P – SF-36 pain score; SF-36 GH – SF-36 general health score. All scores range between 0 (worst possible score) and 100 (best possible score). GAD-7 score – General Anxiety Disorder-7 score where 0 corresponds to minimal anxiety and 21 corresponds to severe anxiety. PHQ-9 score – Patient Health Questionnaire-9 score where 0 corresponds to minimal depression and 27 corresponds to severe depression.

Supplementary Table S2. Change in different quality-of-life measure scores between baseline and the 15-month follow-up.

	**Mean difference in the control group (SE)[a]**	**Mean difference in the intervention group (SE)**	**Total difference (95% CI); Indep. samples t-test P-value****	**Mann-Whitney U test value; P-value**
EQ-5D-5L questionnaire				
EQ-5D-5L scale	-0.021 (0.008)	0.02 (0.008)	0.041 (0.02 to 0.06); P<**0.001**	P<**0.001**
EQ VAS score	-0.30 (0.70)	1.41 (0.74)	1.71 (-0.3 to 3.7); P=0.093	P=**0.014**
EQ-5D-5L mobility score	0.067 (0.038)	-0.06 (0.041)	-0.13 (-0.24 to -0.018); P=**0.023**	P=**0.044**
EQ-5D-5L self-care score	0.062 (0.027)	0.06 (0.029)	-0.01 (-0.08 to 0.07); P=0.88	P=0.752
EQ-5D-5L usual activities score	-0.003 (0.037)	0.02 (0.039)	0.02 (-0.09 to 0.13); P=0.73	P=0.984
EQ-5D-5L pain/ discomfort score	0.062 (0.043)	-0.17 (0.043)	-0.23 (-0.35 to -0.11); P<**0.001**	P<**0.001**
EQ-5D-5L anxiety/ depression score	0.006 (0.040)	-0.12 (0.041)	-0.13 (-0.24 to -0.015); P=**0.026**	P=**0.021**
MTBQ questionnaire				
MTBQ scale	1.17 (0.53)	0.83 (0.48)	-0.34 (-1.8 to 1.1); P=0.64	P=0.59
MTBQ w/o 9-10 scale	1.33 (0.55)	1.00 (0.52)	-0.33 (-1.8 to 1.1); P=0.66	P=0.46
MTBQ item 1	0.014 (0.040)	-0.05 (0.040)	-0.06 (-0.17 to 0.05); P=0.30	P=0.15
MTBQ item 2	0.050 (0.037)	0.00 (0.035)	-0.05 (-0.15 to 0.05); P=0.32	P=0.41
MTBQ item 3	0.094 (0.051)	0.003 (0.036)	-0.09 (-0.22 to 0.03); P=0.14	P=0.61
MTBQ item 4	0.081 (0.029)	0.15 (0.032)	0.07 (-0.02 to 0.15); P=0.12	P=**0.014**
MTBQ item 5	0.13 (0.031)	0.01 (0.034)	-0.12 (-0.21 to -0.03); P=**0.010**	P=**0.002**
MTBQ item 6	0.11 (0.042)	0.14 (0.048)	0.03 (-0.1 to 0.15); P=0.68	P=0.33
MTBQ item 7	0.089 (0.058)	0.03 (0.055)	-0.06 (-0.21 to 0.10); P=0.50	P=0.47
MTBQ item 8	0.072 (0.050)	0.20 (0.055)	0.12 (-0.02 to 0.27); P=0.10	P=0.11
MTBQ item 9	0.10 (0.041)	0.04 (0.031)	-0.06 (-0.16 to 0.041); P=0.24	P=0.57
MTBQ item 10	0.081 (0.037)	0.12 (0.037)	0.03 (-0.08 to 0.13); P=0.63	P=0.008
MTBQ item 11	0.097 (0.028)	0.06 (0.025)	-0.04 (-0.11 to 0.04); P=0.30	P=0.35
MTBQ item 12	-0.028 (0.047)	0.04 (0.051)	0.07 (-0.07 to 0.21); P=0.31	P=0.35
MTBQ item 13	0.053 (0.027)	0.013 (0.024)	-0.04 (-0.11 to 0.031); P=0.27	P=0.22
IPA questionnaire				
IPA AI	-0.051 (0.032)	-0.06 (0.029)	-0.01 (-0.10 to 0.07); P=0.79	P=0.71
IPA FR	-0.016 (0.034)	-0.05 (0.030)	-0.04 (-0.12 to 0.05); P=0.44	P=0.58
IPA AO	-0.087 (0.037)	-0.11 (0.033)	-0.02 (-0.12 to 0.08); P=0.66	P=0.43
IPA SLR	-0.042 (0.031)	-0.04 (0.027)	-0.002 (-0.08 to 0.08); P=0.96	P=0.77
IPA WE	-0.027 (0.030)	-0.12 (0.034)	-0.10 (-0.19 to -0.007); P=**0.034**	P=**0.029**
SF-36 questionnaire				
SF-36 PF	-1.08 (0.93)	0.26 (0.89)	1.34 (-1.2 to 3.9); P=0.30	P=0.094
SF-36 RLPH	1.77 (1.91)	2.64 (2.08)	0.87 (-4.7 to 6.4); P=0.76	P=0.30
SF-36 RLEP	-0.86 (1.953)	-2.17 (2.04)	-1.31 (-6.9 to 4.2); P=0.64	P=0.58

SF-36 EF	0.00 (0.747)	2.95 (0.82)	2.95 (0.77 to 5.1); P=**0.008**	P=**0.039**
SF-36 EW	-2.25 (0.811)	0.55 (0.78)	2.79 (0.58 to 5.0); P=**0.013**	P=0.072
SF-36 SF	0.40 (0.969)	0.50 (0.98)	0.10 (-2.6 to 2.8); P=0.94	P=0.93
SF-36 P	1.00 (1.824)	4.65 (1.57)	3.64 (-1.1 to 8.4); P=0.13	P=0.16
SF-36 GH	-0.23 (0.630)	1.76 (0.66)	1.99 (0.2 to 3.8); P=**0.030**	P=**0.011**
GAD-7 score	0.32 (0.17)	-0.94 (0.19)	-1.27 (-1.8 to -0.8); P<**0.001**	P<**0.001**
PHQ-9 score	0.42 (0.17)	-0.41 (0.18)	-0.82 (-1.3 to -0.3); P<**0.001**	P<**0.001**

SE – standard error. [a] The mean difference was calculated as the difference between the values at the 15-month follow-up and values in the beginning of the study. ** The total difference was calculated as (Mean difference in the intervention group)-(Mean difference in the control group); the significance of the difference between the groups was evaluated with the independent-samples t-test and the Mann-Whitney U test. EQ-5D-5L scale – values from EQ-5D-5L utility value scale where -0.59 corresponds to the worst possible life quality state and 1.00 corresponds to the best life quality state. EQ VAS score – values of the EQ visual analogue scale ranging between 0 (worst self-rated health score) and 100 (best self-rated health score). EQ-5D-5L M – EQ-5D-5L mobility score; EQ-5D-5L SC – EQ-5D-5L self-care score; EQ-5D-5L UA – EQ-5D-5L usual activities score; EQ-5D-5L PD – EQ-5D-5L pain/discomfort score; EQ-5D-5L AD – EQ-5D-5L anxiety/depression score. All scores range between 1 (no associated problems) and 5 (extreme problems/unable to carry out the activity). MTBQ score – values from MTBQ numeric scale ranging between 0 (no treatment burden) and 100 (maximum treatment burden) calculated as mean(MTBQ questionnaire item values) × 25. MTBQ item 1-MTBQ item 13 – scores for MTBQ questionnaire items 1-13 ranging between 0 (“not difficult/no burden”) and 4 (“extremely difficult/extreme burden”). Item 1 – “Taking lots of medications”, item 2 – “Remembering how and when to take medication”, item 3 – “Paying for medications and treatment”, item 4 – “Collecting prescription medication”, item 5 – “Monitoring your medical conditions”, item 6 – “Arranging appointments with health professionals”, item 7 – “Seeing lots of different health professionals”, item 8 – “Getting time off work, arranging transport, etc. to see doctors”, item 9 – “Getting health care in the evenings and at weekends”, item 10 – “Getting help from community services”, item 11 – “Obtaining clear and up-to-date information about your condition”, item 12 – “Making recommended lifestyle changes”, item 13 – “Having to rely on help from family and friends”. IPA AI – IPA autonomy indoors score; IPA FR – IPA family role score; IPA AO – IPA autonomy outdoors score; IPA SLR – IPA social life and relationships score; IPA WE – IPA work and education score. All scores range between 0 (“very good” response) to 4 (“very poor” response). SF-36 PF – SF-36 physical functioning score; SF-36 RLPH – SF-36 role limitations due to physical health score; SF-36 RLEP – SF-36 role limitations due to emotional problems score; SF-36 EF – SF-36 energy/fatigue score; SF-36 EW – SF-36 emotional well-being score; SF-36 SF – SF-36 social functioning score; SF-36 P – SF-36 pain score; SF-36 GH – SF-36 general health score. All SF-36 scores range between 0 (worst possible score) and 100 (best possible score). t-test and Mann-Whitney P-values highlighted in bold correspond to statistically significant entries at significance level α=0.05.

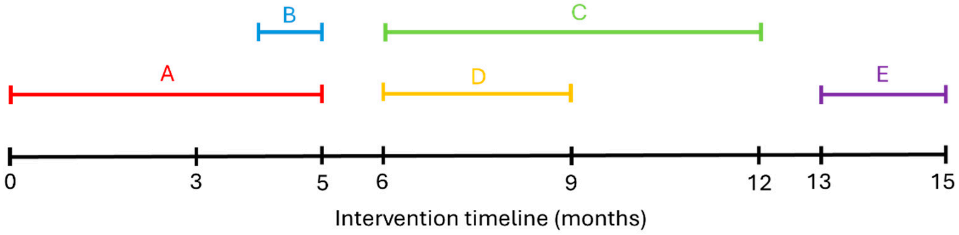

Supplementary Figure S1. Intervention timeline. The intervention group received a more extensive examination and care than is common in typical clinical practice over a span of 15 months. The intervention consisted of a holistic assessment of patient health and the creation of a personalized healthcare plan by the patient case manager and a primary care physician (months 1-5, A), an additional patient care assessment by a board of primary care specialists (months 4-5, B), the execution of the holistic healthcare plan (months 6-12, C), additional personalized expedited need-based examinations (months 6-9, D) and an ultimate assessment of the success in executing the holistic personalized healthcare plan (months 13-15, E).

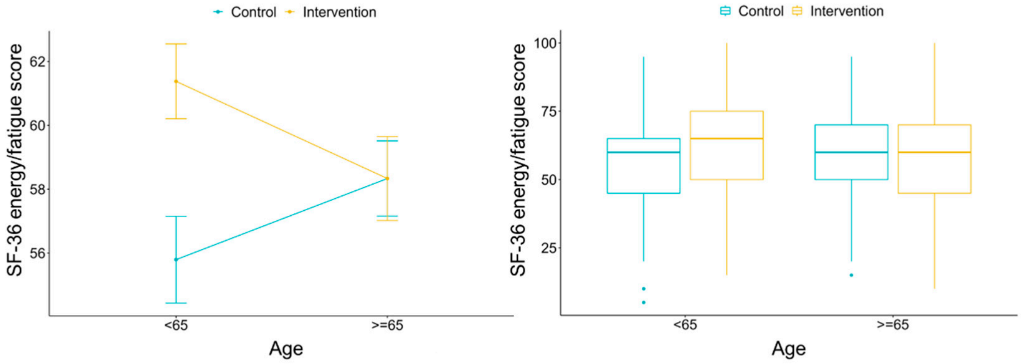

Supplementary Figure S2. Example distribution of different health outcome scores that are statistically significantly different among control and intervention group patients in different categories of sociodemographic or clinical factor variables. Images on the left – mean and standard error values, images on the right – boxplots with highlighted median, interquartile range, ±1.5 × IQR values and outliers. Control group – blue, intervention group – gold. The statistical significance of the differences in distributions was calculated with ANOVA and ART ANOVA methods, results are reported in Table 3. Only the distributions that have P<0.05 values for both the ANOVA and ART ANOVA tests are reported in Table 3 and in this figure.
